# A cross-sectional study of breast pain in a diverse population of breast cancer patients

**DOI:** 10.1097/or9.0000000000000055

**Published:** 2021

**Authors:** Jami Fukui, Erin Bantum, Madison Meister, Shannon Lim, Ashley Davidson Marumoto, Ian Pagano

**Affiliations:** a University of Hawaii Cancer Center, 701 Ilalo Street, Honolulu, HI 96813, USA; b University of Louisville 2310S. Third Street, Louisville, KY 40292, USA; c Hawaii Residency Programs, 1356 Lusitana Street, Honolulu, HI 96813, USA

**Keywords:** Breast cancer, Breast pain, Racial/ethnic disparities

## Abstract

**Background:**

Breast cancer is the most common cancer in women, and postoperative breast pain has been reported to be anywhere from 25% to 60%. However, there is sparse data regarding racial/ethnic differences in breast pain among breast cancer patients.

**Methods:**

We performed a cross-sectional anonymous survey of breast cancer patients from the Hawaii Cancer Consortium over a 6-week period between 2019 and 2020. The 237 breast cancer participants were ages 29 to 98, 74% Asian, and mainly from outpatient oncology clinics. We evaluated the prevalence of breast pain in a diverse group of breast cancer patients and characterized the pain using a modified short-form McGill pain questionnaire (sfMPQ).

**Results:**

Eighty-fourrespondents(35.4%) reported breast pain. On univariable analysis, we found significant racial/ethnic differences in the amount of breast pain, where Chinese and Japanese participants reported significantly less pain compared to White participants on a 10-point pain scale. We found differences in breast pain according to age and endocrine therapy use as well as survey location, however, no differences were seen according to chemotherapy, radiation, or breast surgery. Based on the sfMPQ, the most common descriptors of breast pain were sensory (throbbing, shooting, and stabbing) compared to affective (tiring-exhausting, sickening, fearful, and punishing-cruel) characteristics. Although they were described as mild and intermittent, in women with breast pain, 33.4% reported the breast pain affected their sleep, 16.7% their work, and 15.4% their sexual activity.

**Conclusions:**

Breast pain is a significant problem in our breast cancer community. This survey assessment has informed our understanding of breast pain in our diverse population. In turn, we are developing culturally appropriate pain management strategies to treat this challenging symptom common in breast cancer survivors.

## Introduction

Breast cancer is the most common cancer in women.^[1]^ Breast pain is a common symptom experienced by breast cancer survivors and often refers to postsurgical pain which can become chronic. Prevalence rates for persistent pain following breast cancer surgery are reported for up to 60% of patients.^[2–5]^ Younger age, use of radiotherapy, more invasive surgery, and acute postoperative pain have been identified as predictors of persistent pain after breast cancer surgery.^[2,4,6–9]^ Racial disparities in cancer treatment-related symptom burden are linked to worse treatment outcomes.^[10]^ Black breast cancer patients report more pain, symptom intensity, and decrease in physical functioning and distress with breast cancer treatments compared to White women.^[3,11,12]^ There are also reported differences in physical, social, and emotional well-being among Black cancer survivors compared to White, where Black survivors reported less physical and social well-being but better emotional well-being.^[13]^ Black and Latina cancer patients have been found to have poorer pain management compared to White cancer patients.^[14]^ In addition, provider communication and decision-making experience were worse in Latina breast cancer patients compared to White.^[15]^ Poorly managed symptoms can result in cancer treatment delays and nonadherence, with negative implications for survival.^[10]^ Routine symptom assessment for all breast cancer survivors, as well as clinicians’ management of symptoms for racially diverse cancer patients, need to be more thoroughly studied and addressed.

Several studies have looked at breast pain in breast cancer survivors, specifically in factors predicting postoperative and persistent breast pain, but to the best of our knowledge, none of these studies have reported on differences between ethnic groups.^[16]^ A study performed in China found 28.5% of women reported persistent pain after surgery and 50.5% of the people reported sensory disturbance, while 4.2% reported phantom breast pain.^[17]^ The ethnic population of Hawaii is diverse. Hawaii has no racial/ethnic majority group, based on population size. About half of the population is of Asian heritage (Japanese, Filipino, Korean, and Chinese), about a quarter is of European ancestry (White), and about 20% Native Hawaiian.^[18]^ Given the breast cancer symptom burden experienced particularly in ethnic minority groups and the paucity of data in breast cancer patients with breast pain, we investigated the prevalence of breast pain and its associated factors, in addition to characterizing breast pain in a diverse population of breast cancer patients in Hawaii. Understanding the differences of breast pain for our diverse breast cancer population can inform future interventions aimed at improving equity in symptom management.

## Methods

### Study population

We conducted a cross-sectional anonymous survey of breast cancer patients from the Hawaii Cancer Consortium over a 6-week period between 2019 and 2020. Surveys were offered in 4 areas: outpatient oncology clinics, breast cancer survivorship groups, social work in-person visits, and as an online link. Patients were eligible for the study and were given the opportunity to participate if they 1) had a diagnosis of breast cancer, 2) were English-speaking, and 3) had not completed the survey at a prior time.

### Study procedures

Eligible patients were approached on arrival by a front-desk staff person or social worker. Interested participants were given the anonymous paper survey or paper flier with the online link. Completed paper surveys were returned directly to front-desk staff or social workers and were picked up weekly by the research team. Patients could decline the paper survey or flier, although there were limited refusals.

### Measures

Demographic variables included age and self-reported ethnicity, whereas cancer status included various indicators of the current diagnosis and treatment status (breast cancer diagnosis, type of surgery-lumpectomy or mastectomy, number of lymph nodes removed, use of radiation, chemotherapy, and/or endocrine treatment). The breast pain questionnaire was based on the short-form McGill pain questionnaire (sf-MPQ)^[19]^ and consisted of 25 questions. The McGill Pain Questionnaire (MPQ) is a self-report questionnaire, consisting of 3 major classes of word descriptors—sensory, affective/emotional impact, and cognitive evaluation of pain.^[20]^ The sfMPQ was developed to provide an instrument that could be completed in less time than the MPQ but would still reflect both the sensory and affective dimensions of pain^[19]^ and has been shown to have high correlations with the original McGill Pain Scale. Breast pain was assessed using multiple variables, including quality, intensity, amount, pattern, duration, timing, location, and associated aggravating or alleviating factors. In addition, if pre-menopausal, patients were asked if there was any association with the menstrual cycle, and all patients were asked if breast pain affected work, sleep, or sexual activity. Participants were asked to also discuss any other pain either associated with breast pain or not and if any medications or other modalities were used to relieve breast pain.

### Statistical methods

We summarized data as frequencies and percentages and ran linear regression to model associations. In multivariable analyses, we included all study variables as covariates to adjust for confounding, and we also provide univariable (unadjusted) results for comparison. The outcome was breast pain (0–9 scale) with predictors age, ethnicity, location of the survey, surgery, radiation, chemotherapy, endocrine therapy, and lymph nodes. We chose the simplest relevant groupings for the predictor variable categories. The analytic methods are for a cross-sectional study with simple sampling, addressing missing data by creating a missing category for each predictor. We did not run analyses by subgroups or interactions. Assuming a Type I error rate (alpha) of 0.05, and medium effect size (Cohen f equal to 0.25 or 6% of the variance explained), 53 patients per group provide 80% power. We considered *P*-values <.05 to be statistically significant. The SAS 9.4 software (SAS Institute In, Cary, NC, USA) performed all analyses.

## Results

### Sample characteristics

**Figure 1.**
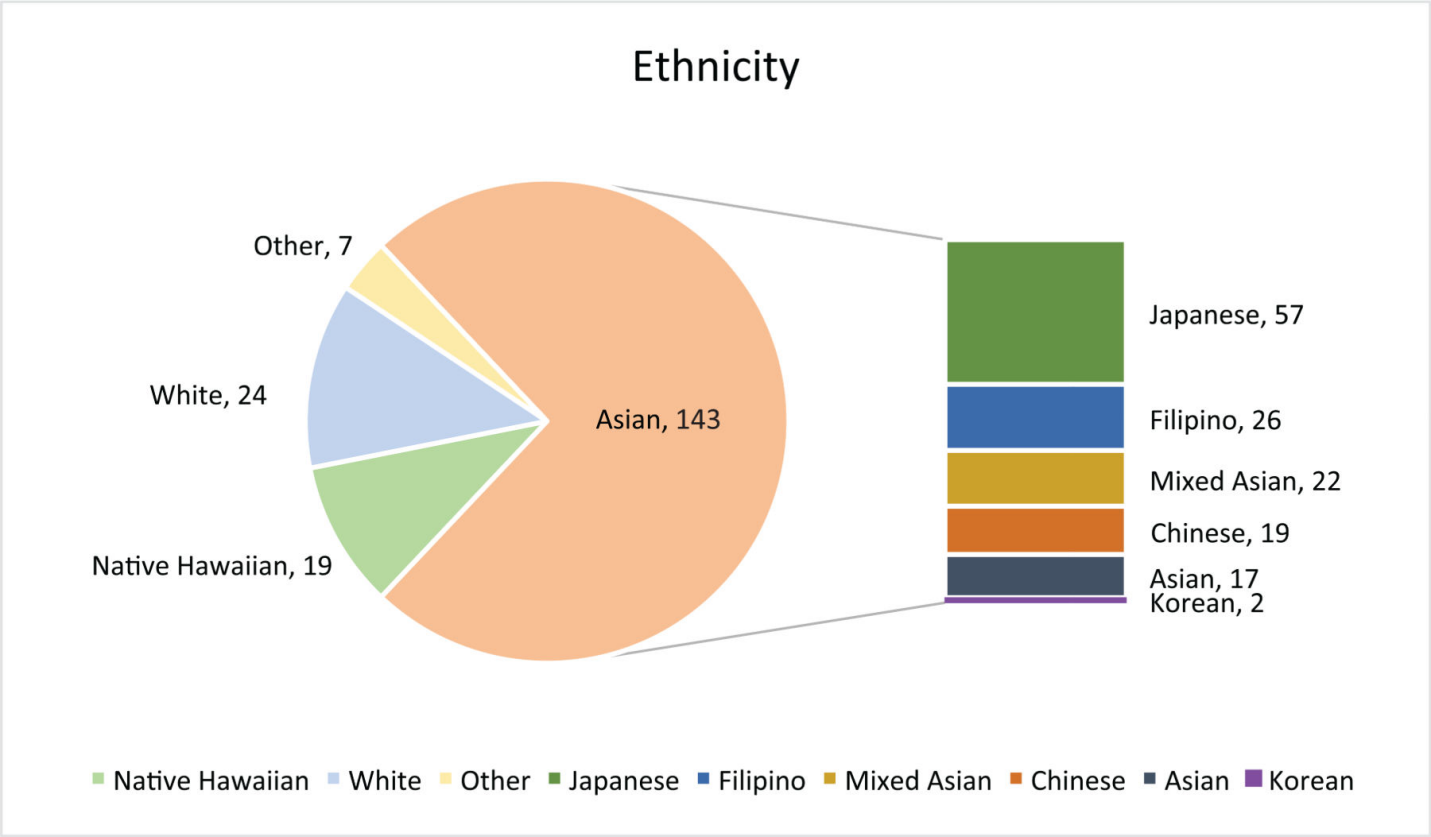
Racial/ethnic make-up of survey respondents.

A total of 237 questionnaires were collected and analyzed. A significant percentage of participants were older than 60years of age (n=118, 49.8%), with 25.3% (n=60) being older than 70 years old (Table 1). About 87.4% identified as non-White (n=169), with the majority identifying as Asian (n=143, 74%) followed by White (n=24, 12.4%) and Native Hawaiian (n=19, 9.8%) (Fig. 1). A 86.9% (n=206) of the sample completed the assessment in paper format which included oncology clinics, social work visits, and support groups. Slightly more participants underwent breast conservative surgery (n=110, 46.4%), compared to (n=88, 37.2%) patients who had a mastectomy. Participants in this study commonly had 1 to 3 lymph nodes removed (n=90, 38%). Correlating with the surgical procedure, radiation was more common (n=150, 63.6%) compared to no radiation therapy (n=64, 27%). More study participants received chemotherapy (n=113, 47.7%) than those who did not (n=86, 36.3%), suggesting a slightly higher risk breast cancer population. Endocrine therapy use was common (n=143, 60.3%), consistent with the known prevalence of hormone receptor-positive subtype of breast cancer and its adjuvant treatment recommendations.

### Demographics

In the univariable regression analysis (Table 2), we found significant racial/ethnic differences in the amount of breast pain, where Chinese and Japanese participants reported significantly less pain compared to White participants on a 10-point pain scale. The youngest age group (18–39) and age group 70 to 79 reported more breast pain than other aged participants on multivariable analysis. Participants receiving endocrine therapy had higher breast pain, and although not statistically significant, those currently receiving radiation reported lower breast pain.

### Pain characteristics

**Figure 2.**
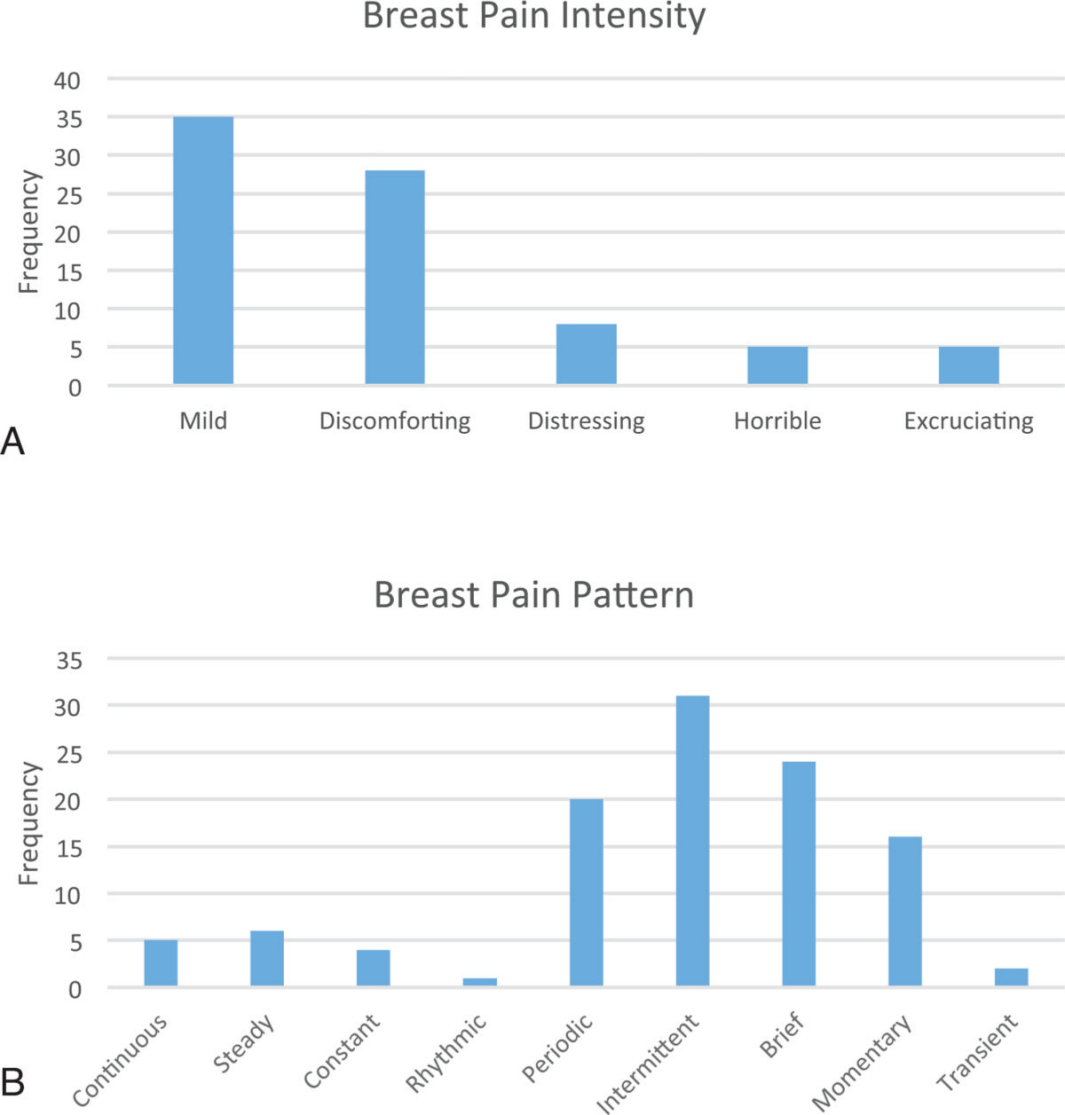
Present pain intensity and pattern. (A) Overall breast pain intensity was described as mild (n=35, 41.7%). (B) Most common pattern of breast pain: intermittent, brief, periodic, and/or momentary (n=91).

Eighty-four respondents (35.4%) reported breast pain, where 35 participants (n=35, 41.7%) reported a 3 or 4/10 pain level and 43% described overall breast pain as mild, based on the present pain intensity 6-point Likert scale: 0=no pain, 1=mild, 2=discomforting, 3=distressing, 4=horrible, and 5=excruciating (Fig. 2A). The temporality of pain was mainly described as intermittent, brief, periodic, and/or momentary (n=91; Fig. 2B).

**Figure 3.**
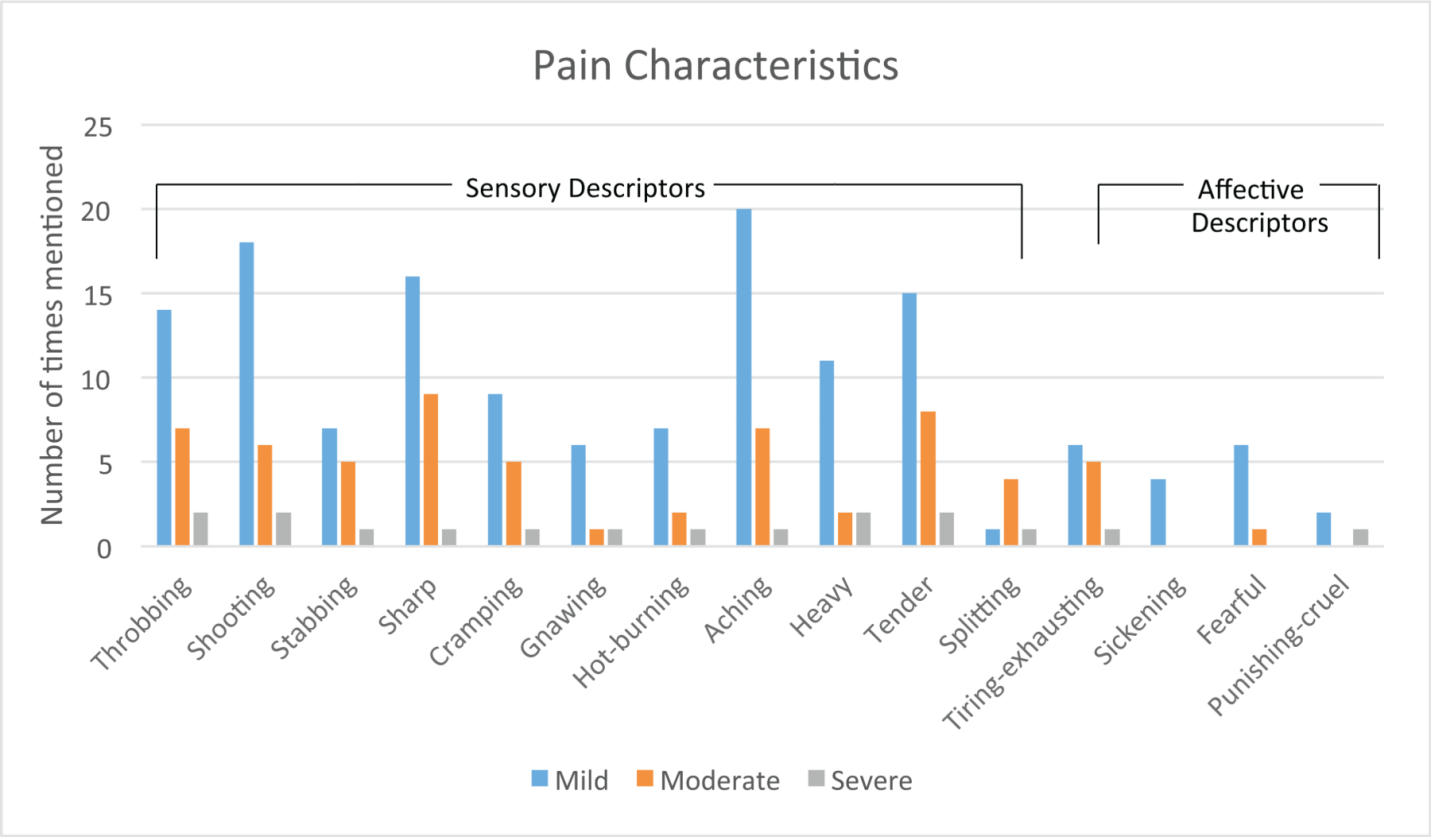
Breast pain characteristics. Overall, the most common descriptors of breast pain were from sensory qualities of pain compared to affective pain quality descriptors. For mild pain; aching, for moderate pain; sharp and there was not a most common descriptor for severe pain.

The most common descriptors of breast pain were from sensory qualities of pain (throbbing, shooting, stabbing, sharp, gnawing, cramping, hot, aching, heavy, tender, and splitting; Fig. 3). The effective pain quality descriptors were less common (tiring-exhausting, sickening, fearful, and punishing-cruel; Fig. 3).

For mild pain, the most common descriptor was aching (n=20), for moderate pain the most common descriptor was sharp (n=9), and was a mix for severe pain—heavy, tender, shooting, and throbbing (n=2) without a most common descriptor (Fig. 3).

**Figure 4.**
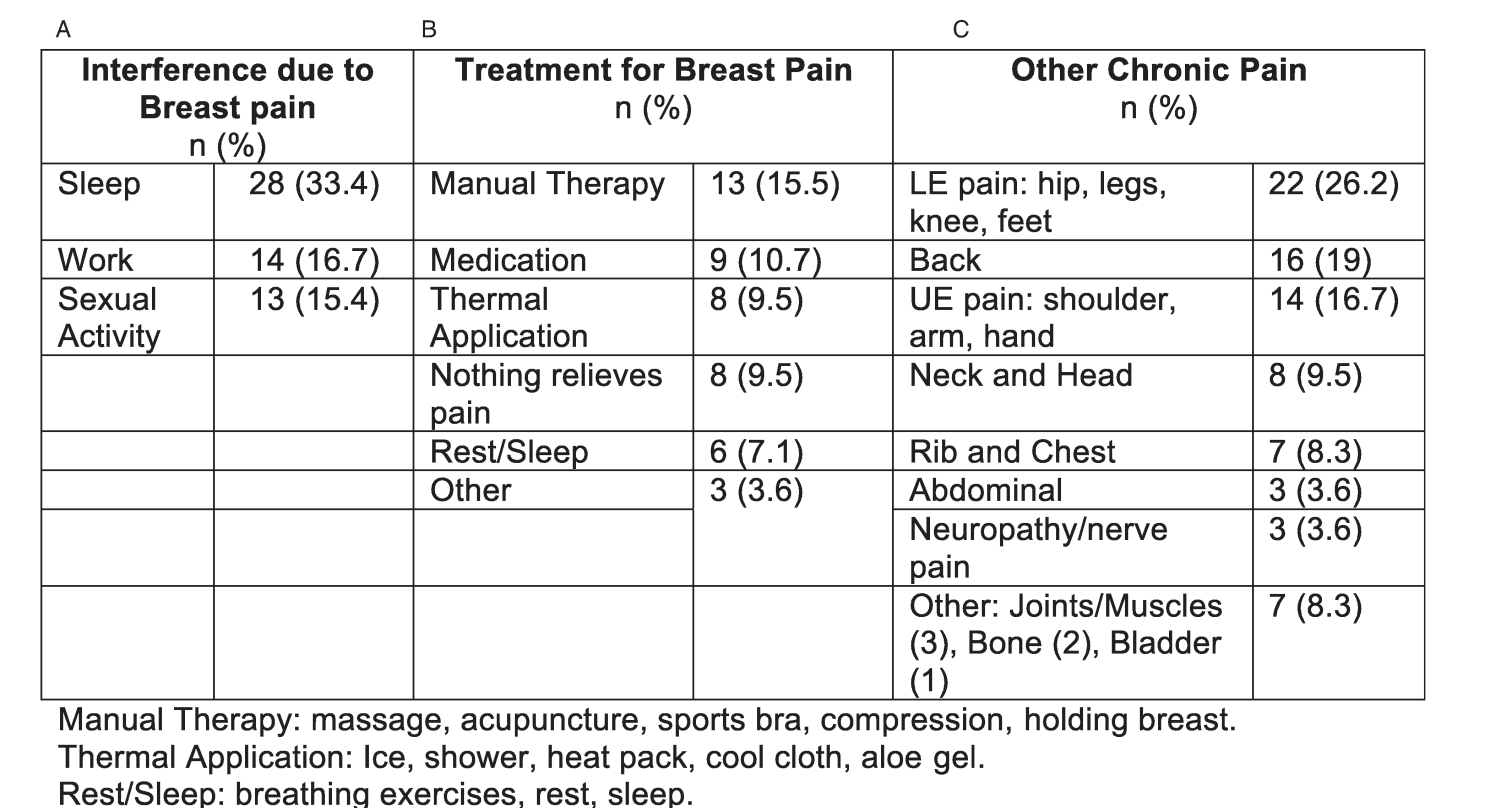
(A) Breast pain interference, (B) treatment, and (C) other chronic pain.

Twenty-eight patients (33.4%) reported breast pain affected their sleep with 16.7% (n=14) reporting it affected their work and 15.4% (n=13) reporting it affected their sexual activity (Fig. 4A). The most common treatment modalities reported to help relieve breast pain were manual therapy (n=13, 15.5%), followed by medication (n=9, 10.7%), and thermal application (n=8, 9.5%), although there were a variety of responses (Fig. 4B). A majority of participants reported other chronic pain, most commonly lower extremity: hip, legs, knee, and feet (n=22, 26.2%), followed by back (n=16, 19%) and upper extremity: shoulder, arm, and hand (n=14, 16.7%) (Fig. 4C).

## Discussion

In this cross-sectional study of breast cancer patients at the Hawaii Cancer Consortium, there were significant racial/ethnic differences in the amount of breast pain reported. Some of our findings elucidate nuances that provide guidance and differ from existing literature. We found that sensory qualities of pain were more commonly reported than affective characteristics overall, which according to the sfMPQ include: throbbing, shooting, stabbing, sharp, gnawing, cramping, hot, aching, heavy, tender, and splitting, and the affective pain quality descriptors include: tiring-exhausting, sickening, fearful, and punishing-cruel. These various qualities of pain help to identify characteristics of pain and suggest potential treatments which can address these symptoms uniquely. For example, a descriptor that falls within the group category of sensory, such as tender or splitting, are characteristics of neuropathic type pain and may be more amenable to neuropathic directed treatments with therapeutics such as gabapentin. Whereas the descriptor sharp which is also sensory but in the incisive pressure group is characteristic of mechanical pain and may be more responsive to direct physical manipulation with the massage. Similarly, effective pain quality descriptors may indicate an emotional component to the pain and suggest psychologically focused treatment.

Some complementary and integrative therapies have been found to be efficacious for the treatment of cancer pain. Due to the multimorphism of cancer pain, certain mind-body therapies such as massage, acupuncture, healing touch, hypnosis, and music therapy can help to address anxiety, stress, depression, or mood disturbances. Other therapies such as yoga, tai chi/qigong, guided imagery, virtual reality, and cognitive-behavioral therapy alone or combined, have shown trends in reducing the severity of cancer pain.^[21]^ In several recent randomized controlled trials, hypnosis positively influenced pain, distress, fatigue, and nausea.^[22]^ The mindfulness-based intervention has been found efficacious in reducing persistent pain in women treated for breast cancer.^[23]^ There remains a need for research of current psychotherapeutic interventions and their efficacy and the role of mediator variables (eg, coping) on pain perception in cancer patients.

The “breast pain” term refers to pain in the breast that is unrelated to the type of surgery (ie, postmastectomy pain syndrome or duration of pain. Chronic postsurgical pain is defined as chronic pain that develops or increases in intensity after a surgical procedure or a tissue injury and persists beyond the healing process, that is, at least 3months after the surgery or tissue trauma.^[24]^ It is under-recognised and often undertreated and is not represented in the current International Classification of Diseases (ICD-10). Recently the International Association for the Study of Pain has reclassified chronic postsurgical pain for the update in ICD-11 to improve the identification, diagnosis, and treatment of these pain states.^[24]^

There are literature describing racial/ethnic differences noted in the literature in regards to other types of pain. Some studies have evaluated chronic musculoskeletal pain systematically and found differences in pain beliefs, cognitions, and behaviors in patients from different racial backgrounds.^[25]^ Some have hypothesized that Asian cultures view the inability to tolerate pain as a weakness^[26]^ which may contribute to the lower pain value noted in most races other than White in our population. Other studies show a variable range in pain threshold and tolerance among different ethnic groups.^[17]^ The way in which ethnicity interacts with the setting in which pain is expressed should also be considered, in addition to the inherent differences regarding the settings in which people are most likely to report pain. This could include patient-provider communication, available time to discuss concerns, personality factors, and the impact of pain on current functioning.

Breast pain differences were seen according to some treatments in our study, specifically those who received endocrine therapy, where we did see an increase in breast pain and the likelihood of having breast pain, which has also been noted in a recent review evaluating common breast pain treatments.^[27]^ For example, breast density is a known risk factor for breast cancer development and survival^[28]^ where younger women are more likely to have dense breasts compared to older women^[29]^ and breast density in Asian women compared to other races is typically higher.^[30,31]^ In our study, we did not measure density, so this could not be confirmed on a case-by-case basis, yet we found Chinese and Japanese women reported less breast pain. Whether this is unique to breast pain or has other driving factors, such as the desire to express pain directly is unknown. Most of the literature discusses breast density and breast pain in the context of hormonal intervention,^[32]^ where there is no change in the incidence of breast pain or breast density based on the hormonal intervention studied. A potential mechanism for increased breast pain among younger women could be their inherent increased breast density, however would not explain the racial/ethnic differences seen.

There are some data evaluating genetic polymorphisms which can affect the metabolism of catecholamines and modulate responses to sustained pain. Individuals homozygous for the met158 allele of the catechol-O-methyltransferase (COMT) polymorphism (val158met) showed different responses to pain compared with heterozygotes and were found to have higher sensory and affective ratings of pain.^[33]^ COMT and other potential genetic polymorphisms could influence the pain experience and may underlie racial/ethnic differences seen in the pain response.

### Limitations

All the data collected for this study were self-report, which captures the important experience of the variables of interest. Because pain is dynamic and oftentimes complex, self-report data could inherently have limitations in capturing the extent and characteristics of pain. Our sample included all breast cancer patients irrespective of their current treatment or stage, therefore determining etiologies of pain remains a major limitation. Although the refusal rate to participate in the survey was low, it can contribute to the selection bias of this study. People who opted into joining the study could have a better relationship with their healthcare provider, to include better patient-provider communication, thus leading to more of an openness to communicate about breast pain and/or some pain already being treated. In addition, people might have opted into taking the study if they were experiencing breast pain, which could provide an estimation of breast pain that is higher than the actual estimate. Although different aspects of who opted in and out could impact the findings in a number of different ways that are not entirely clear here, it is important to keep in mind that this selection bias makes exact generalizability to the population impossible.

In addition, the sfMPQ was limited to English-speaking patients and excludes a small but significant percentage of our patient population. The cross-sectional nature of the study precludes us from drawing conclusions regarding the potential impact on pain-related outcomes. Based on the limited data collected, we cannot make any conclusions about different pain treatments and its influence on pain evaluation. In addition, to limit the length of the survey, no social factors were collected and may also confound our data. Despite the study’s limitations, however, the findings provide preliminary data that can lead to suggestions of potential cultural-specific interventions to address breast pain symptoms.

## Conclusions

This study has informed our understanding of the type of pain our diverse breast cancer patients are experiencing and highlighted differences in reported pain by ethnic group, allowing for continued work to understand differences in the way in which pain is experienced and reported in future studies. Our ultimate goal is to develop culturally informed and relevant pain management strategies to treat this challenging symptom for breast cancer patients. This work is an initial step in understanding some of the relevant factors.

## Figures and Tables

**Table 1:** Respondent demographics (n=237).

Variable*		N	%
Age	18–39	13	5.5
	40–49	30	12.7
	50–59	50	21.1
	60–69	58	24.5
	70–79	43	18.1
	80–99	17	7.2
Ethnicity	Asian^†^	19	9.8
	Chinese	19	9.8
	Filipino	26	13.5
	Hawaiian	19	9.8
	Japanese	57	29.5
	Mixed Asian^†^	22	11.4
	Other	7	3.6
	White	24	12.4
Location	Oncology clinic	202	85.3
	Online	31	13.1
	Support group	4	1.7
Surgery type	Lumpectomy	110	46.4
	Mastectomy	81	34.2
	Both	7	3.0
Radiation	No	64	27.0
	Yes	150	63.3
	Current	12	5.1
Chemotherapy	No	86	36.3
	Yes	113	47.7
	Current	28	11.8
Endocrine	No	81	34.2
	Yes	143	60.3
Lymph nodes	0	38	16.0
	1 –3	90	38.0
	4–9	29	12.2
	10+	32	13.5

* Approximately 10% of cases did not state age, radiation, chemotherapy, or endocrine treatment, and approximately 20% of cases did not state race, surgery, or lymph node involvement.

† Self-identified Asian or mixed Asian, no particular country mentioned.

**Table 2:** Continuous breast pain analysis according to respondent demographics, survey location, and treatment.

				Univariable		Multivariable	
Variable		N	%	Mean	*P*	Mean	*P*
Age	18–39	13	5.8	2.65	.001	2.22	.003
	40–49	30	13.3	1.32	.11	1.25	.06
	50–59	49	21.7	1.49	.02	1.02	.12
	60–69	52	23.0	0.57		0.40	
	70–79	40	17.7	1.40	.05	1.60	.004
	80–99	17	7.5	0.44	.83	0.67	.63
	Missing	25	11.1	1.08	.31	2.19	.006
Ethnicity	Asian	19	8.4	1.29	.16	1.42	.39
	Chinese	19	8.4	0.53	.009	0.49	.02
	Filipino	25	11.1	1.32	.14	1.34	.28
	Hawaiian	18	8.0	1.06	.08	1.40	.39
	Japanese	55	24.3	1.01	.02	1.39	.27
	Mixed Asian	20	CO CO	1.50	.27	1.40	.38
	White	24	10.6	2.19		1.94	
	Other	6	2.7	2.17	.98	2.08	.88
	Missing	40	17.7	0.74	.007	0.21	.006
Location	Oncology clinic	192	85.0	0.99	.005	1.01	.02
	Online	30	13.3	2.13		2.02	
	Support group	4	1.8	3.38	.26	3.25	.25
Surgery type	Lumpectomy	103	45.6	0.94		1.13	
	Mastectomy	79	35.0	1.49	.08	1.06	.84
	Both	7	3.1	1.14	.81	0.72	.62
	Missing	37	16.4	1.20	.52	1.66	.27
Radiation	No	62	27.4	1.37		1.32	
	Yes	143	63.3	1.16	.51	1.04	.39
	Current	10	4.4	0.00	.06	0.26	.14
	Missing	11	4.9	1.45	.90	3.04	.09
Chemotherapy	No	81	35.8	1.16		1.30	
	Yes	108	47.8	1.37	.50	1.39	.73
	Current	27	11.9	0.67	.29	0.80	.29
	Missing	10	4.4	0.70	.52	−1.02	.03
Endocrine	No	75	33.2	0.75		0.75	
	Yes	139	61.5	1.39	.03	1.34	.05
	Missing	12	5.3	1.42	.31	1.99	.08
Lymph nodes	0	38	16.8	0.93		0.98	
	1 –3	84	37.2	1.26	.43	1.26	.48
	4–9	28	12.4	1.70	.15	1.35	.45
	10+	32	14.2	1.55	.22	1.56	.27
	Missing	44	19.5	0.66	.55	0.83	.76
